# The influence of change on sense of coherence on dental services use among adolescents: a two-year prospective follow-up study

**DOI:** 10.1186/s12903-021-02026-9

**Published:** 2021-12-25

**Authors:** Carlos Augusto da Silva Araújo Júnior, Janete Maria Rebelo Vieira, Maria Augusta Bessa Rebelo, Fernando José Herkrath, Ana Paula Corrêa de Queiroz Herkrath, Adriana Corrêa de Queiroz, Juliana Vianna Pereira, Mario Vianna Vettore

**Affiliations:** 1 School of Dentistry, Federal University of Amazonas, Av. Ministro Waldemar Pedrosa, 1539, Praça 14 de Janeiro, Manaus, AM CEP 69025-050 Brazil; 2 Instituto Leônidas e Maria Deane, Fundacão Oswaldo Cruz, Manaus, Rua Teresina, 476, Adrianópolis, Manaus, AM CEP: 69027-070 Brazil; 3 Department of Health and Nursing Sciences, Faculty of Health and Sports Sciences, University of Agder, Campus Kristiansand, Universitetsveien 25, 4630 Kristiansand, Norway

**Keywords:** Dental health services, Sense of coherence, Adolescents

## Abstract

**Background:**

To investigate the influence of change on sense of coherence (SOC) on dental services use in adolescents over a two-year period.

**Methods:**

A prospective follow-up study was conducted involving 334 12-year-old adolescents from public schools in the city of Manaus, Amazonas, Brazil. The predictors of use of dental services in the last 12 months were selected according to the Andersen’s behavioural theoretical model. The predisposing factors included sex, self-reported skin colour and SOC. The enabling factors were dental insurance, monthly family income and parents/guardians schooling. Dental pain, perceived oral health status, dental caries and gingival status were used to assess need factors. Multivariable Poisson regression with robust variance was used to estimate incidence-rate ratios (IRR) and 95% confidence intervals between the independent variables and use of dental services.

**Results:**

Adolescent’s SOC scores decreased significantly between baseline and one-year follow-up. SOC decline decreased the likelihood of using dental services in the last 12 months (IRR = 0.96 95%CI 0.92–0.99). Dental caries (IRR = 1.03 95%CI 1.01–1.04) and gingival bleeding (IRR = 1.01 95%CI 1.01–1.02) remained associated with use of dental services in the last 12 months. Adolescents with dental pain were more likely to have visited a dentist in the last year (IRR = 1.03, 95%CI 1.01–1.06).

**Conclusion:**

SOC decrease over one-year period was a meaningful factor of dental services use among 12-year-old adolescents. Dental pain and clinical conditions were also relevant factors that can influence use of dental services in this group.

## Background

The long-term benefits of regular dental attendance on oral health have been shown since routine attenders had less tooth loss and less dental caries than non-routine attenders [1]. Nonetheless, access to dental care is often challenging, representing a public health problem in most countries due to restrictions related to financing and healthcare organization models [2]. The limited access to dental care might influence the oral health status of the population resulting in a substantial burden of dental diseases, including dental caries of deciduous and permanent tooth, periodontal disease and tooth loss [3].

Use of dental services is unevenly observed across the different demographic and socioeconomic population groups [4]. For instance, dental services utilization was lower in less developed countries, in younger children than older children, and among children with unsupportive families (eg. single parent or having no supportive relatives versus married or coupled) [4]. The determinants of dental care use are complex as they result from the interrelationships between socio-environmental characteristics, psychosocial factors, material circumstances, and dental treatment needs [4–7]. Most previous research on this topic has adopted the Andersen’s behavioural model of health services use, which is a conceptual framework of understanding the multiple dimensions of health service use [8, 9]. According to Andersen’s model, the predictors of health services use can be grouped into predisposing characteristics (demographic and psychosocial factors), enabling characteristics (income and health insurance) and need characteristics (perceived health and evaluated needs) [8, 9].

Psychosocial factors reflect the interrelation between social factors and individual’s mind in influencing positive health-related behaviours [10]. Sense of coherence (SOC) is the central construct of the Salutogenic model proposed by Antonovsky. SOC can defined as a global orientation conveying the extent to which one has a pervasive, enduring though dynamic feeling of confidence that one’s internal and external environments are foreseeable and the greater likelihood that forthcoming experiences will occur as reasonably expected [11]. SOC is shaped by life experiences, including consistency, underload-overload balance, and participation in socially valued decision-making. Ultimately, SOC can be interpreted as a psychosocial factor that facilitates people to identify, mobilize and use the available resources effectively to promote coping by finding solutions in a health-promoting manner [11]. The concept of SOC reflects a person’s view of life as structured, manageable, meaningful, or coherent, and the capacity to respond to stressful situations to promote health [12]. Thus, SOC concept is a dispositional orientation rather than a personality trait/type or a coping strategy [13]. SOC was correlated with other standardized measures, including general health, optimism, self-esteem, life events, quality of life and wellbeing [14].

The SOC scale was developed to operationalize the construct using a 29-item scale (SOC-29) or a short form of 13 items (SOC-13) expressing the three components of the construct: comprehensibility (the ability of people to understand what is happening around them), manageability (the extent to which they feel capable of managing the situation) and meaningfulness (the ability to find meaning in the situation). Nonetheless, the instrument is considered unidimensional consisting of one global factor [13, 14]. Previous studies using SOC-13 reported Cronbach’s alpha ranging from 0.70 to 0. 92, and test–retest correlation from 0.79 to 0.71 (1-year interval), suggesting the instrument is reliable [14]. The validity of the SOC scale was considered acceptable or moderate when face, consensual, construct, criterion, and predictive validity were examined [14].

Adolescence is a period of life characterized by physiological and cognitive changes while new tasks and challenges become part of the daily life of the individuals [15]. Several social, emotional and cognitive processes are shaped during this developmental stage that might influence the possibility of the individual to reduce the potential of hazardous and unhealthy behaviours [16]. A stronger SOC enhances adolescent adaptive capacity to respond to challenging situations through the development and expansion of cognitively based strategies [17]. Thus, SOC can be considered a critical resource for effective coping and a meaningful factor to handle stressful circumstances during adolescence. The understanding that SOC plays a central role on adolescents’ health behaviours through moderating stress experiences and favouring health promoting behaviours can be considered the point of departure to hypothesize that SOC might influence the frequency and pattern of dental visits [17]. Individuals with greater SOC are possibly more likely to have positive attitudes towards oral health and thus anticipate the benefits of the dental visits [12, 17].

Some studies have explored the relationship between SOC and use of dental services [5, 7, 18–20]. Swedish adults with stronger SOC were more likely to visit dentists regularly for check-ups [19]. In contrast, strong SOC was linked to less use of dental services in Norwegian adults [7]. Children’s pattern of dental attendance was associated with a stronger maternal SOC in Brazil [5, 18, 20]. Existing evidence on the possible role of SOC in the use of dental services among children and adolescents has important shortcomings that need to be further explored. First, research on this topic is predominantly supported by cross-sectional studies. Thus, previous findings on the association between greater SOC and use of dental services rely on the theoretical assumptions of the conceptual models since cross-sectional studies are unable to demonstrate the temporal relationship between variables. Second, current knowledge suggests that SOC is developed during childhood and adolescence, and the stability of SOC occurs only during adulthood. So, since SOC may vary over time until the adulthood, studies examining the link between SOC and use of dental services in adolescents should assess SOC changes over time instead of measuring SOC in only one-time point. Third, as mentioned above, most of the previous studies assessed the relationship between maternal SOC and children’s utilization of dental services [5, 18, 20]. Therefore, there is a lack of understanding on the possible role of self-reported SOC and utilization of dental services among adolescents. Finally, recent evidence from cluster-randomized controlled trials demonstrated that children’s SOC can be increased following school-based interventions [21, 22].

The aforementioned gaps prompted us to conduct a longitudinal study to evaluate whether adolescent’s SOC changes predicts use of dental services. The present study was designed to evaluate the influence of SOC changes on the use of dental services in 12-year-old adolescents over a period of two years.

## Methods

A prospective follow-up study was conducted in the eastern region of the city of Manaus, Brazil. The region is a socially deprived urban area comprising eleven neighbourhoods. Baseline and follow-up data were collected within a two-year period. The strengthening the reporting of observational studies in epidemiology (STROBE) checklist was used to report the study. Adolescents aged 12 years regularly enrolled in the year 7 in 25 public schools in the East zone of the city were invited to participate. A representative sample of 528 adolescents was obtained through a stratified random sampling process considering the size of schoolchildren population across the neighbourhoods. Of them, 86 did not return the consent form or their parents did not agree their participation (response rate = 83.7%). Of the 442 remaining adolescents, 27 were excluded because of the use of orthodontic appliances. Baseline and follow-up data collection involved 415 and 334 participants, respectively. The power of the study was estimated as 90% considering a sample of 334 adolescents, a significance level of 5%, and a 95% confidence interval to estimate associations of 1.20.

Baseline data were collected from the adolescents and their parents/guardians in 2016. Baseline questionnaire was completed by adolescents to obtain demographic data (sex and skin colour), SOC, dental pain and perceived oral health status in a separate room in the schools. Information on adolescent’s use of dental services, adolescent’s dental insurance, monthly family income and parents/guardians schooling was obtained from the parents/guardians through a self-completed questionnaire. Dental clinical examinations were conducted amongst adolescents in the schools using a plain dental mirror No 5 and a WHO ball-point probe to assess dental caries and gingival status. All adolescents performed oral hygiene under supervision before the dental exams. Follow-up data collection occurred after one year in the schools when the adolescents responded the SOC questionnaire. Participants were lost in the follow-up due to move to another school or to another city despite the attempt to reach them using the telephone contact number obtained at baseline. Use of dental services during the last 12 months was assessed at two-year follow-up.

### Theoretical model

**Fig. 1:**
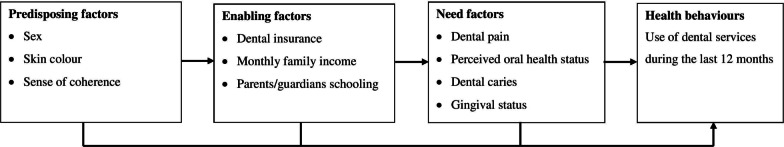
Theoretical model on the predictors of use of dental services adapted from Andersen’s behavioural conceptual model

The theoretical model used in this study was adapted from the Andersen’s behavioural model to assess the association between SOC and use of dental services considering the individual predisposing, enabling and need factors [8] (Fig. 1).

### Outcome

The clinical guideline used in the UK (NICE) recommends that dental recall intervals for people younger than 18 years old should be no longer than 12 months for maintaining good oral health and appropriate home care habits [23]. Thus, use of dental services during the last 12 months (0 = adolescent has not had any dental visit during the last 12 months, 1 = adolescent had at least one dental visit during the last 12 months) assessed at two-year follow-up was the outcome of the study.

### Predictor variables

Predisposing factors were sex (male/female), self-reported skin colour, and SOC. Self-reported skin colour was assessed according to the methodology proposed by the Brazilian Institute of Geography and Statistics (IBGE), that classifies the Brazilian population into five categories: branco (white), pardo (brown), preto (black), amarelo (yellow), and indigenous as standard.

Adolescent SOC was measured using the cross-culturally adapted Brazilian version of the Antonovsky SOC-13 scale [13, 24]. The SOC scale is composed of 13 items measuring comprehensibility (example item: “*When you talk to people, do you have a feeling that they don’t understand you?*” (from ‘never have this feeling’ to ‘always have this feeling’)), manageability (example item: “*When you do something that gives you a good feeling*:” (from ‘it’s certain that you’ll go on feeling good’ to ‘it’s certain that something will happen to spoil the feeling’)) and meaningfulness (example item: *Doing the things you do every day is*: (from ‘a source of deep pleasure and satisfaction’ to ‘a source of pain and boredom’)) [13, 24]. The SOC-13 is responded using a five-point Likert scale, and the final score can range from 13 to 65, which is obtained by summing up the item’s responses. Items related to negative SOC are reversed before calculating the total SOC score. The greater the score, the higher the SOC. The trans-cultural adaptation of SOC-13 into Portuguese language was made into four stages as described elsewhere [24]. The main modifications of the SOC-13 were: (i) the scale was changed into a five-point Likert scale respecting the semantic limits of extreme answers, (ii) The negative question was substituted for its corresponding affirmative form, and (iii) Some rewording was performed to adjust the meaning. For instance, words such as “frequency” and “extremes” were rephrased as they were not well comprehended [24]. The Brazilian version of SOC-13 showed adequate psychometric properties. Cronbach’s alpha of the scale was 0.80 and Spearman’s correlation for test–retest reliability was 0.76 [24].

In this study, adolescent SOC was assessed at baseline and at one year of follow-up. The difference of SOC scores between baseline and one-year follow-up was calculated. Thus, positive values of the difference of SOC scores were interpreted as a decline of SOC, whereas negative values of the difference of SOC scores meant an increase of SOC.

Enabling factors were adolescent dental insurance (1 = yes, 2 = no), monthly family income and parents/guardians schooling. Monthly family income was the sum of income of all residents and was registered in Brazilian minimum wages (BMW) (1 =  ≤ ½ BMW, 2 =  > ½ to 1 BMW, 3 =  > 1 BMW). One BMW corresponded to U$271.09 in 2016. Parents/guardians schooling was assessed according to the total number of years of schooling with approval.

Need factors included dental pain, perceived oral health status, dental caries and gingival status. Dental pain was assessed whether the adolescent experienced toothache in the last 6 months (0 = no, 1 = yes). The perceived oral health status was measured using the Child Perceptions Questionnaire (CPQ_11-14_), consisting of 16 items grouped into 4 dimensions (oral symptoms, functional limitation, emotional state and social well-being) [25]. The total CPQ_11-14_ score may vary from 0 to 64, and the higher the score the worst the perception of oral health. Multi-item instruments comprising different dimensions of perceived health, such as CPQ_11-14_, have a better validity and are more sensitive to assess subjective health-related measures. Dental caries was assessed through the number of decayed, missing and filled teeth index (DMFT index) [26]. Gingival status was evaluated according to the number of teeth with bleeding on probing. All teeth in one upper quadrant and one lower quadrant randomly selected were examined [27].

### Calibration study and reliability during the main study

Five dentists were calibrated for dental examinations previous to data collection of the main study. Twenty adolescents who did not participate in the main study were examined twice. Inter-examiner Kappa coefficient for DMFT ranged from 0.914 and 0.988. During the main study, 10% of the participants were randomly re-examined to assess the reliability of the data. Intra-examiner Kappa coefficient for DMFT was 0.930. The intraclass correlation coefficient of agreement for SOC and CPQ_11-14_ were 0.888 and 0.830, respectively. Cronbach coefficient was 0.674 for SOC and 0.812 for CPQ_11-14_.

### Data analysis

Initially, the predictor variables were compared between participants lost during follow-up and those who completed the 2-years follow-up through Pearson Chi-square test (categorical variables) and *t*-test (continuous variables). The descriptive data was presented through means and proportions, and respective 95% confidence intervals (95% CI). SOC scores between baseline and one-year follow-up were compared using Wilcoxon paired test. Poisson regression with robust variance was used to estimate the incidence-rate ratios (IRR) and 95% CI between predisposing, enabling and need factors and use of dental services. SOC scores significantly decreased between baseline and one-year follow-up. Thus, the difference of SOC scores was considered the main exposure. The regression coefficients of the difference of SOC scores were multiplied by 10 on the log scale, indicating a change in the outcome variable for every increase of 10 units in the difference of SOC. Initially, the Akaike's information criterion (AIC) of the null model with the school-level variable (AIC = 813.53) and without the school-level variable (AIC = 811.53) were estimated through Poisson regression. The differences in use of dental services between schools were non-significant when the null models with and without the school-level variable were compared using likelihood ratio test (*P* = 0.368). Therefore, multilevel analysis accounting for clustering was not used. The association between each independent variable and use of dental services during the last 12 months was assessed through unadjusted Poisson regression. Statistical modelling using multivariable Poisson regression with robust variance was performed according to the Andersen’s behavioural model. Model 1 included the predisposing factors (sex, skin colour and SOC change). The following model (Model 2) included the predisposing factors and the enabling factors (dental health insurance, monthly family income and parents/guardians schooling). The final model (Model 3) was composed of predisposing factors, enabling factors and need factors (dental caries, gingival status, dental pain and perceived oral health status). All independent variables were retained in the final model. All analyses were performed using the STATA® 16.0 program (StataCorp, College Station, Texas, TX, USA).

### Ethical aspects

The present research was approved by the Research Ethics Committee of the Federal University of Amazonas (Protocol Number 57273316.1.0000.5020). All parents signed a written informed consent agreeing with their children’s participation in the current study before data collection.

## Results

The predisposing, enabling and need variables did not differ statistically between participants lost during the follow-up and those who completed the follow-up (Table 1). The characteristics of the sample according to use of dental services in the last 12 months in the follow-up period are presented in Table 2. Of the 334 adolescents who completed the two-year follow-up, 43.4% were males and 68.3% had pardo (brown) skin colour. In the two-year follow-up period, 58.4% (95% CI 53.0–63.6) of the adolescents reported at least one dental visit during the last 12 months. The means of SOC score at baseline and one-year follow-up were 45.8 and 44.2, respectively. On average, SOC score decreased by 1.6 (95% CI 0.8–2.4) over the one-year period. Nearly 90% of the adolescents who completed the 2-years follow-up did not have dental health insurance, and around 40% were from families with a monthly income between > ½ and 1 minimum wage. The mean of parents/guardians years of schooling was 10.3 years. The mean of DMFT and gingival bleeding was 0.9 and 3.4, respectively. CPQ_11-14_ mean score was 14.5, and 36% of the adolescents reported dental pain in the last year (Table 1).

**Table 1:** Predisposing, enabling and need factors between adolescents lost during follow-up and those who completed the 2-years follow-up

Variable	Adolescent lost during follow-up (N = 82)	Adolescents who completed follow-up (N = 334)	*P* value
	N (%)/ Mean (SD)	N (%)/ Mean (SD)	
**Predisposing**			
Sex, N (%)			0.318^b^
Male	30 (37.0)	145 (43.5)	
Female	52 (63.0)	189 (56.5)	
Skin colour, N (%)			0.807^b^
Branco (White)	13 (16.0)	48 (14.4)	
Preto (Black)	5 (6.2)	31 (9.3)	
Amarelo (Yellow)	4 (4.9)	13 (3.9)	
Pardo (Brown)	55 (66.7)	228 (68.2)	
Indigenous	5 (6.2)	14 (4.2)	
SOC, mean (SD)	45.6 (7.7)	45.8 (6.4)	0.771^c^
**Enabling**			
Dental health insurance, N (%)			0.835^b^
Yes	8 (10.0)	36 (10.8)	
No	74 (90.0)	298 (89.2)	
Monthly Family income, N (%)			0.749^b^
≤ ½ BMW^a^	22 (26.0)	91 (27.2)	
> ½ to 1 BMW	30 (37.0)	134 (40.2)	
> 1 BMW	30 (37.0)	109 (32.6)	
Paternal schooling, mean (SD)	14.7 (19.6)	10.3 (7.4)	0.052^c^
**Needs characteristics**			
DMFT, mean (SD)	0.7 (1.2)	0.9 (1.5)	0.211^c^
Number of teeth with gingival bleeding, mean (SD)	2.9 (2.9)	3.4 (3.1)	0.164^c^
OHRQoL, total score mean (SD)	14.8 (7.9)	14.5 (9.0)	0.759^c^
Dental pain, N (%)			0.977^b^
Yes	29 (35.8)	120 (36.0)	
No	53 (64.2)	213 (64.0)	

^a^*BMW* Brazilian minimum wage

^b^chi-square test

^c^*t*-test

**Table 2:** Predisposing, enabling and need factors according to the use of dental services in the last 12 months at two-year follow-up among adolescents

Variable	Total baseline (N = 415)	Baseline data among adolescents who completed the 2-years follow-up (N = 334)	Dental visit in the last 12 months at 2-years follow-up (N = 334)	
			Yes	No
	%/Mean (CI 95%)	%/ Mean (CI 95%)	%/ Mean (CI 95%)	%/ Mean (CI 95%)
**Predisposing**				
Sex, %				
Male	42.1 (37.4–46.9)	43.4 (38.2–48.8)	42.6 (35.8–49.6)	44.6 (36.5–53.0)
Female	57.7 (52.9–63.4)	56.5 (51.2–61.8)	57.4 (50.4–64.2)	55.4 (47.0–63.5)
Skin colour, %				
Branco (White)	14.7 (11.6–18.4)	14.4 (11.0–18.46)	13.3 (9.2–18.9)	15.8 (10.6–22.9)
Preto (Black)	8.7 (6.3–11.8)	9.3 (6.6–12.9)	8.2 (5.1–13.0)	10.8 (6.6–17.2)
Amarelo (Yellow)	4.1 (2.6–6.5)	3.9 (2.3–6.6)	4.6 (2.4–8.7)	2.9 (1.1–7.5)
Pardo (Brown)	68.0 (63.1–72.1)	68.3 (63.1–73.1)	69.7 (62.9–75.8)	66.2 (57.9–73.6)
Indigenous	4.6 (2.9–7.1)	4.2 (2.5–7.0)	4.1 (2.1–8.0)	4.3 (1.9–9.3)
SOC, mean	45.8 (45.1–46.4)	45.8 (45.1–46.5)	–	–
SOC difference between baseline and one-year follow-up, mean	–	–	1.9 (0.5–3.4)	4.4 (3.2–5.7)
**Enabling**				
Dental health insurance, %				
Yes	10.6 (8.0–14.1)	10.8 (7.8–14.7)	9.0 (5.2–15.3)	12.0 (8.1–17.5)
No	89.4 (85.9–92.0)	89.2 (85.3–92.2)	91.0 (85.0–95.0)	88.0 (83.0–92.0)
Monthly Family income, %				
≤ ½ BMW^a^	26.9 (22.9–31.5)	27.2 (22.7–32.3)	24.4 (18.8–31.0)	31.2 (24.0–39.4)
> ½ to 1 BMW	39.6 (39.4–44.4)	40.2 (35.1–45.6)	42.0 (35.2–49.1)	37.7 (29.9–46.1)
> 1 BMW	33.5 (29.1–38.2)	32.6 (27.8–37.9)	33.7 (27.3–40.7)	31.2 (24.0–39.4)
Paternal schooling, mean	11.2 (10.1–12.2)	10.3 (9.5–11.1)	10.3 (9.3–11.3)	10.3 (9.0–11.7)
**Needs characteristics**				
DMFT, mean	0.8 (0.7–0.9)	0.9 (0.7–1.1)	1.0 (0.8–1.2)	0.7 (0.5–0.9)
Number of teeth with gingival bleeding, mean	3.3 (3.0–3.6)	3.4 (3.1–3.7)	3.7 (3.2–4.1)	3.0 (1.5–3.5)
OHRQoL, total score mean	14.5 (13.7–15.3)	14.5 (12.9–17.0)	14.3 (13.1–15.5)	14.6 (3.0–15.5)
Dental pain, %				
Yes	35.9 (31.4–40.7)	36.0 (30.9–41.3)	35.6 (29.1–42.6)	36.6 (28.8–45.1)
No	64.1 (59.3–68.6)	64.0 (58.7–69.1)	64.4 (57.4–70.9)	63.4 (54.9–71.2)

^a^*BMW* Brazilian minimum wage

The results of the unadjusted Poisson regression between the independent variables and use of dental services are shown in Table 3. The SOC decrease between baseline and one-year follow-up (IRR = 0.95), DMFT (IRR = 1.03), gingival bleeding (IRR = 1.01) and dental pain (IRR = 1.03) were statistically associated with use of dental services in the last 12 months at 2-year follow-up.

**Table 3:** Unadjusted Poisson regression between predisposing, enabling and need factors and use of dental services in the last 12 months at two-year follow-up among adolescents

Variables	IRR^a^ (95% CI)
**Predisposing**	
Sex	
Male	1
Female	1.01 (0.95–1.08)
Skin colour	
Branco (White)	1
Preto (Black)	0.98 (0.85–1.14)
Amarelo (Yellow)	1.10 (0.92–1.31)
Pardo (Brown)	1.04 (0.94–1.14)
Indigenous	1.02 (0.84–1.23)
SOC change between baseline and one-year follow-up	0.93 (0.90–0.97)*
**Enabling**	
Dental health insurance	
Yes	1
No	0.95 (0.86–1.06)
Monthly family income	
≤ ½ BMW^b^	1
> ½ to 1 BMW	1.06 (0.97–1.15)
> 1 BMW	1.05 (0.96–1.15)
Paternal schooling	1.00 (1.00–1.00)
**Need factors**	
DMFT	1.03 (1.01–1.04)*
Number of teeth with gingival bleeding	1.01 (1.01–1.02)*
OHRQoL	1.00 (0.99–1.01)
Dental pain	
No	1
Yes	1.03 (1.01–1.06)*

^*^P < 0.05

^a^*IRR* Incidence-rate Ratio

^b^*BMW* Brazilian minimum wage

Table 4 presents the multivariable Poisson analysis on the association between SOC change and use of dental services at 2-year follow-up adjusted for predisposing, enabling and need variables. The decline of SOC over one year decrease the likelihood of using dental services in the last 12 months (IRR = 0.96, 95%CI 0.92–0.99). Dental caries (IRR = 1.02, 95%CI 1.01–1.04) and gingival bleeding (IRR = 1.01, 95%CI 1.01–1.02) remained associated with use of dental services in the last 12 months. Adolescents with dental pain were more likely to have visited a dentist in the last 12 months (IRR = 1.03, 95%CI 1.01–1.06).

**Table 4:** Multivariable Poisson regression between predisposing, enabling and need factors and use of dental services in the last 12 months at two-year follow-up among adolescents

	Model 1	Model 2	Model 3
Variable	IRR^a^ (95% CI)	IRR^a^ (95% CI)	IRR^a^ (95% CI)
**Predisposing**			
Sex			
Male	1	1	1
Female	1.01 (0.94–1.08)	1.00 (0.93–1.07)	0.99 (0.93–1.07)
Skin colour			
Branco (White)	1	1	1
Preto (Black)	0.97 (0.84–1.12)	0.96 (0.83–1.11)	0.95 (0.82–1.11)
Amarelo (Yellow)	1.07 (0.90–1.27)	1.05 (0.88–1.26)	1.07 (0.91–1.28)
Pardo (Brown)	1.02 (0.93–1.13)	1.02 (0.92–1.12)	1.02 (0.92–1.12)
Indigenous	1.02 (0.84–1.24)	1.00 (0.82–1.22)	0.97 (0.81–1.17)
SOC change between baseline and one-year follow-up	0.93 (0.90–0.97)*	0.95 (0.92–0.99)*	0.96 (0.92–0.99)*
**Enabling**			
Dental health insurance			
Yes		1	1
No		0.97 (0.87–1.07)	0.96 (0.87–1.07)
Monthly family income			
≤ ½ BMW^b^		1	1
> ½ a 1 BMW		1.05 (0.96–1.14)	1.04 (0.96–1.13)
> 1 BMW		1.04 (0.95–1.14)	1.05 (0.96–1.14)
Paternal schooling		1.00 (0.99–1.01)	1.00 (0.99–1.00)
**Need**			
DMFT			1.03 (1.01–1.04)*
Number of teeth with gingival bleeding			1.01 (1.01–1.02)*
OHRQoL			1.00 (0.99–1.01)
Dental pain			
No			1
Yes			1.03 (1.01–1.06)*

Model 1: Presdiposing variables

Model 2: Presdiposing + enabling variables

Model 3: Predisposing + enabling + need variables

*P < 0.05

^a^*IRR* Incidence-rate Ratio

^b^*BMW* Brazilian minimum wage

## Discussion

The present study adopted the Andersen’s behavioural model to examine the association of predisposing, enabling and needs factors with use of dental services amongst 12-year-old adolescents during a two-year follow-up. According to our findings, on average, adolescents experienced a decline of SOC during the first year of follow-up. In addition, adolescents with the greatest change of SOC during the first follow-up period were less likely to have visited a dentist in the last 12 months at two-year follow-up. Dental clinical measures, including dental caries and gingival bleeding, and dental pain were also significant predictors of use of dental services in the last 12 months. Thus, our findings suggest that predisposing and need factors were relevant predictors of adolescent dental visit.

Previous evidence highlighted the importance of maternal SOC on children’s use of dental services. Overall, a more favourable pattern of dental attendance was observed among children whose mothers had greater SOC scores than those with mothers with lower SOC [5, 18, 20, 28]. Although the decision regarding adolescent’s use of health services is mainly influenced by their parents, a recent study involving Brazilian adolescents showed that dental pain and dissatisfaction with appearance were relevant determinants of dental services use in this age group [29]. As far as the authors are aware, this is the first follow-up epidemiological study investigating whether changes on adolescent SOC over time was a predictor of use of dental services. The lower use of dental services among adolescents who reported a SOC decline over a one-year period reinforces the importance of SOC on adopting health promoting behaviours. Therefore, our findings reinforce the importance of SOC as a useful internal resource to promote health once individuals with greater SOC have the ability to use the available resources more effectively to deal with stressors (manageability) and to perceive life as meaningful to cope with the environment (meaningfulness) [12, 17]. The salutogenic orientation of SOC towards health is also supported by recent studies showing that a stronger SOC can be a determinant of other oral health practices, including frequency of tooth brushing, dietary habits and smoking habits [28].

SOC is considered a psychosocial construct that explain how people facilitate coping with stressors and promote health through shared components of a wide variety of resistance resources, such as cultural stability, ego-strength and social support [11]. The development of SOC during the first decades of life is related to life experiences, including, consistency, load balance, and participation in shaping outcomes [30]. Therefore, SOC decline among adolescents during the first year of follow-up was a relatively unsurprising finding since SOC tend to vary during adolescence as a result of developmental changes, transitions and challenges [17]. Relevant factors, including parental involvement, psychological factors and quality of life have been acknowledged as important factors associated with adolescent’s SOC and use of dental services [14, 17, 31, 32]. Parental monitoring, focused parenting style and feeling the parents care about them were protective factors of adolescent’s SOC [17, 31]. Moreover, there is a strong correlation between self-esteem, quality of life and SOC [14, 27]. Evidence also suggests the importance of parent participation in decision making of child healthcare services use [32]. The potential influence of parental influences and other psychosocial factors on the relationship between SOC and use of dental services deserves further investigation..

Other predisposing factors (sex and skin colour) investigated in this study were not associated with the use of dental services. Previous studies also did not find a relationship of sex and skin colour with use of dental services [20, 33]. In contrast, male sex and preto (black) skin colour were meaningful predictors of poor use of dental services in Brazilian children and adolescents [5, 34]. There is sound evidence on the importance of dental insurance and socioeconomic status on the use of dental services [4–7, 18, 33]. The lack of association between socioeconomic status and dental services use in our study is possibly due to participants homogeneity regarding socioeconomic status since almost 70% of sample was composed of adolescents from low-income families.

In this study, dental pain, dental caries and gingival status were associated with use of dental services irrespective of sociodemographic factors. Even though nearly 16% of the sample never had a dental visit at baseline, this result suggests that socioeconomic characteristics were not relevant determinants of dental services use inequalities among socially deprived adolescents [5]. Our findings are supported by previous studies that reported that poor dental status are relevant elements of dental services use. Dental caries predicted use of dental services in adults and pattern of dental attendance in children [18, 20].

The present study has some limitations that should be acknowledged. Only 12-year-old adolescents who lived in a socially disadvantaged area were investigated. Therefore, our findings should not be generalised to other age groups and adolescents from other socioeconomic levels. In addition, possible changes of other participant’s characteristics between baseline and two-years follow-up, such as family income and dental clinical status, were not considered in the study.

Future studies should investigate populations from different socioeconomic backgrounds and other age groups. The influence of dental caries and gingivitis on dental services use suggests that preventive and health promotion actions should be developed to improve adolescent’s oral health and consequently reduce the burden on dental services. Previous studies indicate that SOC can be enhanced through interventions in schools [21, 22]. Thus, schools may be considered as an important setting to develop strategies aiming at diminishing the dental services use inequalities via improving student’s SOC. Nonetheless, psychosocial intervention studies aiming to assess whether improving adolescent’s SOC could also promote their use of dental services are needed.

## Conclusion

Our results suggest that besides clinical oral conditions, the decline of SOC over one-year period was an important predictor of poor use of dental services among adolescents after two years of follow-up. Adolescence is a critical period related to physical, cognitive, social and emotional development that are important elements of SOC enhancement. School interventions aiming to enhance SOC may also improve dental services use in adolescents since SOC seems unstable in adolescents. However, future intervention studies are necessary to assess the possible impact of SOC improvement on use of dental services in adolescents.

## Data Availability

The datasets used and/or analysed during the current study are available from the corresponding author on reasonable request.
